# The Universal Soldier: Enzymatic and Non-Enzymatic Antioxidant Functions of Serum Albumin

**DOI:** 10.3390/antiox9100966

**Published:** 2020-10-09

**Authors:** Daria A. Belinskaia, Polina A. Voronina, Vladimir I. Shmurak, Mikhail A. Vovk, Anastasia A. Batalova, Richard O. Jenkins, Nikolay V. Goncharov

**Affiliations:** 1 Sechenov Institute of Evolutionary Physiology and Biochemistry, Russian Academy of Sciences, pr. Torez 44, 194223 St. Petersburg, Russia; paulina291731@gmail.com (P.A.V.); vladimir.shmurak@gmail.com (V.I.S.); batalova.phys@gmail.com (A.A.B.); ngoncharov@gmail.com (N.V.G.); 2 Centre for Magnetic Resonance, St. Petersburg State University, Universitetskij pr., 26, 198504 St. Petersburg, Russia; m.vovk@spbu.ru; 3 Leicester School of Allied Health Sciences, De Montfort University, The Gateway, Leicester LE1 9BH, UK; roj@dmu.ac.uk; 4 Research Institute of Hygiene, Occupational Pathology and Human Ecology, bld.93 p.o. Kuz’molovsky, 188663 Leningrad Region, Russia

**Keywords:** albumin, antioxidant enzymes, non-enzymatic antioxidant molecules, allosteric modulation

## Abstract

As a carrier of many biologically active compounds, blood is exposed to oxidants to a greater extent than the intracellular environment. Serum albumin plays a key role in antioxidant defence under both normal and oxidative stress conditions. This review evaluates data published in the literature and from our own research on the mechanisms of the enzymatic and non-enzymatic activities of albumin that determine its participation in redox modulation of plasma and intercellular fluid. For the first time, the results of numerous clinical, biochemical, spectroscopic and computational experiments devoted to the study of allosteric modulation of the functional properties of the protein associated with its participation in antioxidant defence are analysed. It has been concluded that it is fundamentally possible to regulate the antioxidant properties of albumin with various ligands, and the binding and/or enzymatic features of the protein by changing its redox status. The perspectives for using the antioxidant properties of albumin in practice are discussed.

## 1. Redox System in Health and Disease: Brief Overview

The production of reactive oxygen species (ROS) and reactive nitrogen species (RNS) is an inherent property of all tissues. ROS and RNS play a significant role in the regulation of the main functions of cells: they participate in the reactions of oxidative phosphorylation, transmission of intracellular signals from various growth factors, modulation of various transcriptional proteins, prostaglandin biosynthesis, mitosis and several other processes [1,2].

The sources of ROS in cells are well known. The NAD(P)H-oxidase system (NOX) is perhaps the foremost since ROS production is its main function. In the inflammatory and immune response, NOX produces a superoxide anion by electron transfer from NAD(P)H to molecular oxygen [3,4]. NOX1 and NOX2 isoforms promote the development of endothelial dysfunction, hypertension and inflammation. NOX2 acts as the main source of skeletal muscle ROS during contractions [5]. NOX4 is the only isoform that generates hydrogen peroxide instead of superoxide radical [3]. The investigation of NOX functions has not lost its relevance in 2020 in the context of the COVID-19 pandemic. Thus, in the research of Violi et al. [6], it has been demonstrated that oxidative stress caused by NOX2 activation is associated with severity of the disease and thrombotic events in COVID-19 patients.

Mitochondria are a powerful source of cellular ROS and contain a number of enzymes that convert molecular oxygen to superoxide or its derivative hydrogen peroxide [7,8]. Because of the leak of electrons from the mitochondrial enzyme complexes I and III, about 2–5% of molecular oxygen is converted into the active form. Moreover, monoamine oxidase and cytochrome-β5 reductase in the outer mitochondrial membrane, glycerol-3-phosphate dehydrogenase and cytochrome P450 in inner mitochondrial membrane, and matrix enzymes aconitase, pyruvate dehydrogenase and α-ketoglutarate dehydrogenase can produce the superoxide radical [3]. Currently, long-chain fatty acid dehydrogenase (LCAD) and very long-chain fatty acid dehydrogenase (VLCAD) are discussed as candidates to be added to the list of mitochondrial ROS generators [8].

NO synthases (NOS) are heme-containing proteins that catalyse the conversion of L-arginine to L-citrulline and NO, with the help of FAD, FMN and tetrahydrobiopterin (BH4). There are three main isoforms of the enzyme: neuronal (nNOS), which functions primarily in the central and peripheral nervous system, but also in skeletal muscles; inducible (iNOS), expressed in many cell types in response to cytokines; and endothelial NOS (eNOS), functioning in vascular endothelial cells [9]. BH4 serves as a modulator of NOS activity, providing an additional electron during the catalytic cycle. In its absence, the enzyme produces less NO but more superoxide radical. Ca^2+^ is an activator of NOS: all isoforms can bind calmodulin (CaM), which causes conformational changes in the enzyme molecule, facilitating the reaction [10]. NOS suppression and the resulting NO deficiency play a key role in endothelial dysfunction in different pathologies: infections; cardiovascular and lung diseases; renal and kidney disfunction [11].

Xanthine reductase is another source of ROS in cells [12]. It is a molybdenum-containing enzyme that catalyses the final stage of purine oxidation in humans and the oxidative transformation of pteridines and some aldehydes. Xanthine reductase exists in two forms: xanthine dehydrogenase and xanthine oxidase (XO), which is a post-translational modification of the former. Xanthine oxidase converts molecular oxygen to superoxide anion or hydrogen peroxide [3]. As XO is a major contributor to oxidative stress, the enzyme inhibitors are actively studied as therapeutic agents in pathologies accompanied by oxidative stress development [13,14].

Cytochrome-dependent oxygenases (CYP450) are localised in the smooth endoplasmic reticulum of liver cells, producing a superoxide radical during the oxidation or reduction in a number of endogenous compounds (cholesterol, vitamins, steroids, arachidonic acid) and xenobiotics [15]. Some CYP450s operate in other tissues as well—for example, in cells of the cardiovascular system [3]. Currently, the efforts of many researchers are aimed at studying the role of CYP450 in drug–drug interactions and drug–disease interactions [16,17,18].

We have listed just a few of the most basic sources of ROS and RNS in animal cells. Such systems as animal heme-dependent peroxidases, cyclooxygenase and lipoxygenases, hemoglobin and red blood cells could be added to the list. These systems are described in detail in the reviews [3,19,20,21].

**Figure 1:**
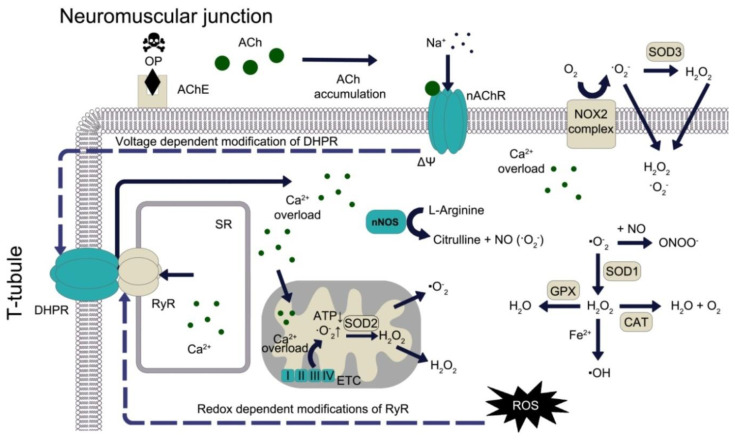
Role of reactive oxygen species (ROS) in the mechanism of toxic effects of organophosphates on skeletal muscle. OP—organophosphate; ACh—acetylcholine; AChE—acetylcholinesterase; nAChR—nicotinic acetylcholine receptor; nNOS—neuronal nitric oxide synthases; ATP—adenosine triphosphate; SOD—superoxide dismutase; ETC—electron transport chain in mitochondria; DHPR—Dihydropyridine receptor; RyR—Ryanodine receptor; SR—Sarcoplasmic reticulum; NOX—NADPH oxidase; GPx—Glutathione peroxidase; CAT—Catalase.

ROS and RNS play an important role in the development of many pathologies: oncological, neurological, cardiovascular, respiratory diseases; pathology of the kidneys and liver; diabetes; intoxications with exogenous xenobiotics of various nature [3,22,23,24,25]. Of special importance is the mechanism of ROS involvement in the toxic effect of organophosphates (OPs) in neuromuscular synapses. Inhibition of acetylcholinesterase (AChE) activity by OPs leads to the accumulation of acetylcholine (ACh) in the neuromuscular synapse, which in turn leads to hyperactivation of nicotinic acetylcholine receptors (nAChR) on the post-synaptic membrane (Figure 1). The principal consequences of excessive activation of nAChR are the uncontrolled entry of sodium ions into the cell and prolonged depolarisation of the plasma membrane. A voltage-dependent conformation change in dihydropyridine receptors (DHPR) coupled to ryanodine receptors (RyR) occurs. This conformational change in the DHPR-RyR complex causes the release of Ca^2+^ from the sarcoplasmic reticulum (SR) into cytosol, which in turn causes contraction of the skeletal muscle. In an uncontrolled release of calcium (in particular, as a result of calcium-induced calcium release) and inability of Ca-ATPase to pump out the abundance of Ca^2+^ ions into the SR, seizures occur.

An increase in the concentration of intracellular calcium leads to the activation of calcium-dependent proteases and mitochondrial enzymes (pyruvate dehydrogenase, isocitrate dehydrogenase and alpha-ketoglutarate dehydrogenase). Seizures require a great amount of ATP, but when electron transport and ATP synthesis are disrupted, an excess quantity of ROS is produced, primarily **∙**O_2_^−^ [26]. Mitochondrial superoxide dismutase 2 (SOD2) converts two superoxide radicals to hydrogen peroxide, and both of these ROS can leave the mitochondrion. The generation of H_2_O_2_ also occurs during the two-electron reduction in oxygen on the mitochondrial electron transport chain (ETC). H_2_O_2_ is a natural uncoupling agent of the ETC: by decreasing the generation of ROS on the ETC, hydrogen peroxide acts as a negative feedback regulator [27].

In addition, calcium overload enhances the work of NOXs, mainly NOX2. NOX2 produces superoxide anion, which is converted into hydrogen peroxide by extracellular superoxide dismutase 3 (SOD3) [28]. **∙**O_2_^−^ and H_2_O_2_ can then re-enter the intracellular space through the chlorine channels and aquaporins [29]. Moreover, Ca^2+^ activates nNOS, which normally produces NO, but in the case of uncoupling, generates superoxide anion [3]. In the intracellular space, the superoxide anion binds to NO to form peroxynitrite. Cytoplasmic superoxide dismutase 1 (SOD1) converts two superoxide radicals into an oxygen molecule and hydrogen peroxide: the latter is destroyed by a catalase (CAT) or glutathione peroxidase (GPX) cycle [30,31]. On the other hand, hydrogen peroxide can be converted to a hydroxyl radical through the Fenton reaction with the participation of Fe^2+^ cations [32].

ROS accumulation in the intracellular space leads to a number of undesirable consequences, including a redox-dependent modification of RyR, which leads to an even greater release of calcium from SR, an increase in calcium overload and seizures. OPs have a similar effect on the heart and respiratory muscles, except that in the heart muscle, DHPR and RyR do not contact each other, and Ca^2+^ ions first enter the cytosol through DHPR, and then Ca^2+^-dependent release of calcium occurs from the sarcoplasm through RyR [33]. By a similar mechanism—via muscarinic receptors, calcium channels and eNOS—OPs lead to endothelial dysfunction, which plays a key role in the pathogenesis of poisoning [34].

The content of active species in cells is strictly controlled by the antioxidant defence system, which include both enzymatic and non-enzymatic processes. The most important non-enzymatic reaction of radical cleavage is their interaction with low-molecular-weight antioxidants such as β-carotene, vitamin C, vitamin E, uric acid, cysteine, glutathione (GSH), polyphenols, etc. As a result of this interaction, the cascade of free radical formation is broken [35].

SOD and CAT, as well as peroxidases, glutathione reductase (GR), glutathione-S-transferase (GST), peroxiredoxin (Prxs), thioredoxin system and paraoxonase (PON) are traditionally included into the enzymatic antioxidant system. These enzymes are widely described in the literature [36,37]. Briefly, SOD (EC 1.15.1.1) converts two superoxide radicals into an oxygen molecule and hydrogen peroxide. CAT (EC 1.11.1.6) catalyses the utilisation of hydrogen peroxide to form molecular oxygen. Peroxidases (EC 1.11.1.x) are a large group of enzymes that catalyse oxidation reactions according to the general scheme: ROOR’+ electron donor (2e^−^) + 2H^+^ → ROH + R’OH. In particular, glutathione peroxidase (GPx, EC 1.11.1.9) ensures the destruction of hydrogen peroxide and lipid hydroperoxides with GSH oxidation. Glutathione reductase (GR, EC 1.8.1.7) reduces oxidised glutathione (GSSG) with the participation of NADPH. A significant role in cellular redox-dependent processes belongs to the family of glutathione-S-transferases (GST, EC 2.5.1.18), which catalyse the conjugation of GSH with a wide range of xenobiotics, weakening their toxic effect [38]. Peroxiredoxins (Prxs, EC 1.11.1.15) control the level of cytokine-induced peroxides involved in cellular signaling. Thioredoxins (Trx) and glutaredoxins (Grx) are a family of the proteins that restore disulfide bonds in other oxidised proteins by disulfide exchange, while thioredoxin reductase (TR, EC 1.8.1.9) reduces the pool of oxidised Trx and Grx with the participation of NADPH [39]. Paraoxonase (PON, EC 3.1.8.1) isoform 1 is associated with high-density and, to a lesser extent, with low-density lipoproteins, protecting them from ROS exposure, whereas PON2 is ubiquitously expressed intracellular protein, localised in mitochondria and the endoplasmic reticulum; PON3 is localised both intracellullarly and on high density lipoproteins [40]. PON1 plays a role in the detoxification of OPs by acting as a catalytic scavenger [41]. 

The above is not a complete list. Antioxidant defence is also represented by the enzymes that metabolise the end products of lipid peroxidation (aldehydes, epoxides, alkenes, alcohol), including epoxyde hydrolases (EC 3.3.2.10) and aldose reductase (EC 1.1.1.21) [42]. Formaldehyde dehydrogenase (EC 1.2.1.46) and lactoylglutathione lyase (EC 4.4.1.5) oxidise their substrates to organic acids using GSH as a coenzyme [43]. Quinone reductase (EC 1.6.5.5) provides a two-electron reduction in quinones to dihydroquinones, which prevents the formation of harmful one-electron reduction products—semiquinones; epoxide hydrolase hydrates epoxides to form diols [44]. In addition, aldehyde dehydrogenase (EC 1.2.1.3) oxidises malonic dialdehyde [45]. Hepatic acyl-CoA thioesterase 1 is worth mentioning, since it has been shown to be involved in promoting oxidative capacity through regulation of FA oxidation [46,47].

Oxidative stress is an abnormality of the prooxidant and antioxidant balance, which can be caused by low levels of antioxidants and/or an increase in the concentration of reactive species [48]. This imbalance causes damage to a wide variety of target structures: lipid membranes, free amino acids, polysaccharides, nucleic acids, receptors and transport proteins. The result of this effect is a change in the functional state of a cell, its transformation or death. Currently, oxidative stress is considered as an important pathogenetic link in the development of more than 200 diseases [49]. Blood as a carrier of biologically active compounds is exposed to oxidants to a greater extent than the intracellular environment, but the concentration of antioxidants in plasma is much lower than in cells [50], and it is albumin that plays one of the key roles in the antioxidant defence of the body under normal conditions and in oxidative stress [51,52].

## 2. Structural Characteristics of Albumin and Their Interspecies Features

**Figure 2:**
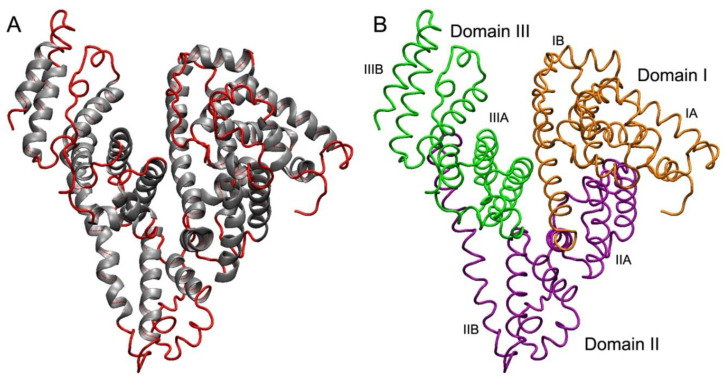
The structure of serum albumin. (**A**)—the secondary structure of albumin: the albumin molecule does not contain β-sheets, α-helices are presented in grey, other regions are shown in red. (**B**)—the tertiary structure of albumin: domains I, II and III are shown in orange, purple and green, respectively; each domain consists of two subdomains (A and B). To create the figure, a three-dimensional structure of human serum albumin from the PDB database, code 3JQZ [57], was used.

We first consider some general information about serum albumin. Albumin is synthesised in the liver at a rate of about 0.7 mg per hour (i.e., 10–15 mg per day); the half-life of human serum albumin (HSA) is about 19–20 days [53]. The molecule of HSA is formed by one polypeptide chain, consisting of 585 amino acid residues. In albumins of other species, the length of the polypeptide chain can vary; in particular, bovine serum albumin (BSA) contains 584 amino acid residues, rat serum albumin (RSA)—583 residues. The secondary structure of the protein contains about 67% helical structures next to 33% of turn and extended chain configurations without any β-sheets [54] (Figure 2A). Three homologous domains (I, II, III), consisting of two subdomains (A, B), form a three-dimensional structure of the protein, which is rather labile (Figure 2B). When albumin interacts with different substances, the effects of cooperativity and allosteric modulation occur, which is more prevalent in multimeric macromolecules [55,56]. 

Many extracellular proteins undergo post-translational glycosylation, which is the process of covalent binding of oligosaccharide chains to amino acid side-chains. In contrast to many other plasma proteins, the albumin molecule is not covered with a carbohydrate moiety under normal conditions, and can bind different endogenous and exogenous ligands: water and predominantly divalent metal cations, fatty acids, hormones, bilirubin, transferrin, nitric oxide, aspirin, warfarin, ibuprofen, phenylbutazone, etc. [58]. Ligand binding occurs at two primary sites (Sudlow sites I and II), which were described for the first time by Gillian Sudlow and co-authors [59]. Additionally, the albumin molecule has the third major binding site (Site III) and several secondary binding centers, the exact number of which is unknown. The albumin molecule contains 17 disulfide bonds and one free thiol group in Cys34. The latter largely determines the participation of albumin in redox reactions. The number of disulfide bonds and Cys34 are conserved in all types of albumin. The role of Cys34 will be discussed in more detail in Section 3.2 and Section 3.3.

The three-dimensional structure of HSA was resolved rather late, only in the 1990s [60]. Previously, it was assumed that the albumin molecule had the shape of an elongated or flattened ellipsoid (“cigar” or “pill”), but X-ray analysis showed that the protein has the shape of a heart. In addition to HSA, three-dimensional structures of BSA [61], albumin of horse and rabbit [61], sheep and goat [62], dogs [63] and cats [64] have been obtained so far.

However, the three-dimensional structure of albumin of rats—the principal animals used in pharmacological and toxicological experiments—has not been obtained yet. Considering the fact that albumin is able to bind almost all known drugs and toxic substances [65,66], this gap should be filled out. The percentage of identity of the primary structures of HSA and RSA is 73.0%, BSA and RSA—69.9%. In some studies, it was shown that HSA and RSA share similar characteristics of binding biologically active substances, but binding efficiencies of some xenobiotics are different for HSA and RSA [67]. Therefore, the correct extrapolation of in vivo results obtained in rats to a human organism requires the identification of amino acids involved in protein–ligand interaction, determination of all structural and conformational features of the binding sites and comparison of the obtained characteristics in HSA vs. RSA. It is especially important for developing of antidotal therapy for OPs poisoning since the use of other mammals in acute experiments is quite complicated. The three-dimensional structure of RSA is needed for such an analysis.

In the absence of crystallographic data, the three-dimensional structure of a protein can be obtained with the help of homologous modeling. The approach allows the construction of a tertiary model of the protein on the basis of its primary sequence and the known three-dimensional structures of homologous proteins [68]. Homologous models of RSA have already been constructed both by our group [67] and other researchers [69].

**Figure 3:**
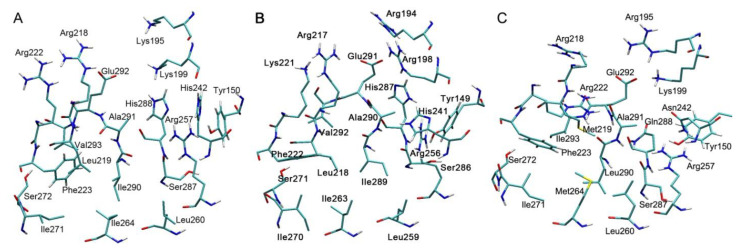
The structures of Sudlow site I of human (**A**), bovine (**B**) and rat (**C**) albumin.

**Figure 4:**
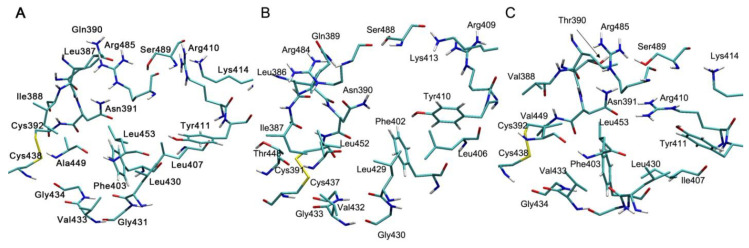
The structures of Sudlow site II of human (**A**), bovine (**B**) and rat (**C**) albumin.

**Figure 5:**
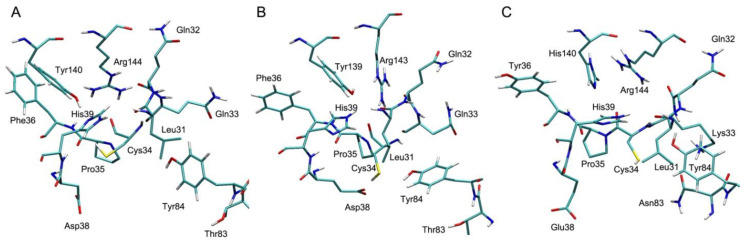
The structures of the redox site (Cys34) of human (**A**), bovine (**B**) and rat (**C**) albumin.

Figure 3, Figure 4 and Figure 5 show the three-dimensional structures of Sudlow site I; Sudlow site II; and the redox site (Cys34) of HSA, BSA and RSA. Due to the deletion at position 116, the numbering of amino acids in BSA after residue 115 is shifted by one position relative to the numbering of HSA and RSA. Below, when discussing the structure of albumins of different species, we give the numbering of HSA as the reference, and if necessary, the corresponding amino acids of BSA is given in brackets—for example, Tyr150(Tyr149). As can be seen in Figure 3 and Figure 4, Sudlow site I is much less conservative than Sudlow site II. Thus, Lys195 and Lys199 of HSA are replaced with more branched-chain arginines Arg194 and Arg198 in BSA. In RSA, Lys195 is also replaced with arginine. Arg222 in HSA and in RSA is substituted by Lys221 in BSA. Leu219 and Leu218 in HSA and BSA are replaced with Met219 in RSA. Similarly, the isoleucines Ile264 and Ile263 in HSA and BSA correspond to Met264 in RSA. Isoleucines are located in the position 290(289) in HSA and BSA, while leucine—in RSA. Valines at position 293(292) in HSA and BSA are replaced with isoleucine in RSA. Histidines His242(His241) and His288(His287) in the primary sequence of HSA and BSA are substituted by Asn242 and Gln288 in the RSA sequence. The latter substitutions are of particular interest since His242(His241) and His288(His287) are located in very close proximity to the catalytic tyrosine Tyr150(Tyr149). According to our computational experiments [70,71,72,73], the imidazole ring of His242(241) can attract the proton of the hydroxyl group of Tyr150(Tyr149) and thus regulate the hydrolytic activity of the tyrosine. It should be expected that interspecies differences in the binding and catalytic properties of albumin will show themselves in the characteristics of Sudlow site I.

Sudlow site II is highly conservative (Figure 4): there are substitutions only in positions 388 (Ile388, Ile387 and Val388 in HSA, BSA and RSA, respectively), 390 (Gln390, Gln389 and Thr390 in HSA, BSA and RSA, respectively), 407 (Leu407, Leu406 and Ile407 in HSA, BSA and RSA, respectively) and 449 (Ala449, Thr448 and Val449 in HSA, BSA and RSA, respectively). All the replacements, except for the homologous substitution at position 407, are located at a sufficient distance from the catalytic tyrosine Tyr411(Tyr410).

Even more surprising differences can be observed in the structure of the redox site near the amino acid residue Cys34 (Figure 5). Gln33, Phe36, Asp38 and Thr83 in HSA and BSA are replaced with Lys33, Tyr36, Glu38 and Asn83 in RSA, respectively. However, the most remarkable difference is that Tyr140(Tyr139) in HSA and BSA is replaced with His140 in RSA. Previously, we showed that Sudlow site I and the redox site of HSA and BSA have a mutual effect on each other: a change in the conformation of one site leads to conformational changes in the other [73,74]. In the redox site, the amino acid residues Cys34, His39, Tyr140(Tyr139) and Arg144(Arg143) and their mutual arrangement (the -SH groups of the cysteine and the -OH groups of the tyrosine relative to the imidazole ring of His39, as well as the -OH group of the tyrosine relative to the side chain of Arg144(Arg143)) play the main role in this effect. How this system works in RSA, where Tyr140(Tyr139) is replaced with a histidine, and how this replacement affects the behavior and availability of Cys34 are still unknown. It is even possible that in rats this mechanism is more effectual than in humans, since these rodents are incredibly omnivorous and adaptable to the environment.

## 3. Albumin Participates in the Redox Modulation of Blood Plasma and Interstitial Fluid

As mentioned above, albumin plays a significant role in the antioxidant defence of the body. The structure of a protein contains a number of amino acids and amino acid sequences that determine its role in redox processes. In this section, we consider three main activities of albumin associated with redox modulation of blood plasma and interstitial fluid.

### 3.1. Binding of Polyvalent Metal Ions

**Figure 6:**
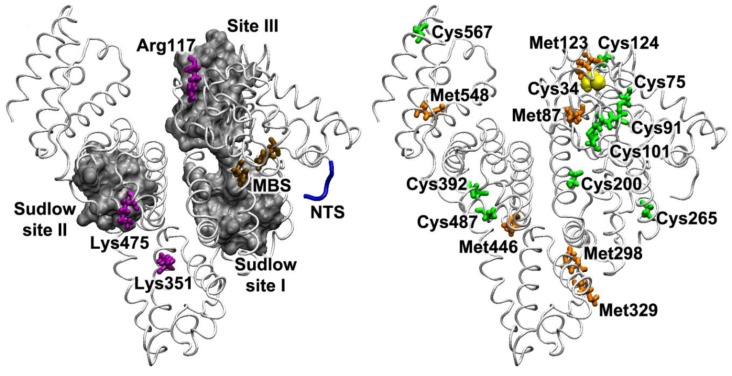
The main binding sites of HSA and the amino acids that are responsible for the participation of albumin in redox reactions. Left: dark gray surface shows the areas of major albumin binding sites—Sudlow site I, Sudlow site II and Site III (see Section 2); the *N*-terminal site (NTS, Asp-Ala-His-Lys) is shown in blue; the metal-binding site (MBS, His-67 and His-247) is shown in brown; the amino acids involved in the binding of polyunsaturated fatty acids are shown in purple. Right: Cys34 is represented in yellow; six methionine residues are shown in orange; the cysteines within the disulfide bonds that can be reduced when interacting with low-molecular-weight thiols are shown in green. To create the figure, a three-dimensional structure of HSA from the PDB database, code 3JQZ [57], was used.

It is well known that polyvalent metals, primarily copper and iron, are pro-oxidants. Copper and iron ions can react with hydrogen peroxide to form toxic hydroxyl radicals (Fenton reaction) [32]. By binding iron and copper cations, albumin heavily reduces their activity: bound ions are still available for reaction, but the radicals formed immediately attack the albumin molecule itself and do not interact with other blood components [75]. In this case, the albumin molecule is damaged, but due to the high concentration of the protein this damage is biologically insignificant. In recent years, some details of the interaction of polyvalent metals with albumin have been determined. Thus, the main binding site for Cu(II) is the *N*-terminal region of human albumin Asp-Ala-His-Lys (*N*-terminal site, NTS) [76] (Figure 6). Binding involves the nitrogen atoms of the backbone and the nitrogen atom of the imidazole ring of the NTS histidine [77]. Using spectroscopic and computational methods, Sendzik et al. [78] showed that the imidazole rings of two histidines play a key role in the binding of the Cu(I) cation. Based on the data obtained, the authors suggested that these histidines can be either His-67 and His-247 of the metal-binding site of albumin (MBS) (Figure 6) or His-3 and His-9 (the first is included in the NTS, the second is in the nearest environment). Normally, albumin is not a physiological carrier of Fe, but it can bind Fe(II) and Fe(III) during pathological iron overload [79]. This binding, however, is apparently non-specific, and takes place somewhere on the surface of the protein and does not involve the NTS or MBS.

### 3.2. ROS Neutralisation

Albumin acts like a ROS trap due to six methionine residues, but mainly due to the free thiol group of Cys34 residue [51,80,81] (Figure 6). This group of activities probably includes the cyanide detoxification reaction by formation of thiocyanate, catalysed in the IIIA subdomain, but without the participation of Tyr411 [82]. In physiological conditions, about 80% of all detected plasma thiols are albumin thiols (99% of GSH is kept in erythrocytes, and about 2/3 of extracellular cysteine/cystine is in a bound form) [83,84]. The Cys34 residue is able to neutralise such ROS and RNS as hydrogen peroxide (H_2_O_2_), peroxynitrite (ONOO^−^), superoxide anion and hypochlorous acid (HOCl), being oxidised to sulfenic acid (HSA-SOH) [51,85].

HSA-SOH is a central intermediate in the processes of redox modulation of blood plasma and interstitial fluid [86]. The final result of the oxidative process depends on what happens next to the sulfenic acid. The HSA-SOH form of albumin can be irreversibly oxidised to sulfinic acid (HSA-S(O)O^−^) [87]. In theory, the side radical of cysteine can be irreversibly oxidised to sulfonic acid (Cys34-S(O)O^−^ O^−^); however, according to the literature, the percentage of Cys34 in the form of sulfonic acid in blood plasma is extremely low [87]. Sulfenic acid can also be converted to disulfide (HSA-S-S-R) by interacting with low-molecular-weight blood plasma thiols (GSH, homocysteine, free cysteine), and then reduced to HSA-SH [88,89,90].

### 3.3. Interaction of Albumin with Low-Molecular-Weight Thiols

Bocedi et al. [89] wanted to answer the question of why a healthy person has only 30 percent of oxidised albumin, while for low-molecular-weight thiols this value is from 70 to 80 percent. The HSA-cysteine conjugate (HSA-Cys34-S-S-Cys) was used as a model of oxidised human albumin, since this disulfide is the main form of oxidised albumin in blood plasma. Biochemical experiments have shown that cysteine is the strongest reducing agent for albumin of the plasma thiols studied, and cystine (cysteine dimer Cys-S-S-Cys) is the strongest oxidising agent. However, it turned out that for the reduction reaction the second order rate constant was about 6 M^−1^∙s^−1^, while for the oxidation reaction it was 10 times less: 0.6 M^−1^∙s^−1^. The authors have concluded that the ratio of the reduced and oxidised forms of albumin is determined by kinetic equilibrium with the cysteine/cystine ratio. In the case of pathology, when the percentage of cystine increases, albumin acts as a redox buffer, maintaining a safe Cys-SH/Cys-S-S-Cys ratio for the body. Moreover, according to the data obtained in this work, the remaining 34 albumin cysteines (forming 17 disulfide bridges) barely undergo cysteinylation even with high concentrations of free cysteine, 150-fold higher than its normal concentration in the blood plasma [89].

According to other experimental data, however, the interaction of HSA with low-molecular-weight thiols involves not only Cys34 but also some cysteine residues that form disulfide bonds: Cys75, Cys90, Cys91, Cys101, Cys124, Cys200, Cys265, Cys392, Cys487, Cys567 [90,91]. Nakashima et al. [90] proposed a mechanism of albumin cysteines thiolation. According to this model, the free thiol group Cys34 is thiolated first. As a result of this reaction, the thiolate anion RS^−^ is formed, which attacks one of the disulfide bonds of albumin. As a result, one of the cysteines that forms disulfide bonds is thiolated, and the second cysteine is converted into the thiolate anion HSA-S^−^ and interacts with the next molecule of low-molecular-weight disulfide, etc. According to the authors’ assumption, the cascade of reactions is interrupted when there are no more disulfide bonds on the surface of the protein available for the thiolate anion. The partial destruction of disulfide bonds of albumin is a rather dramatic event that can lead to protein aggregation and change its functional characteristics.

### 3.4. Enzymatic Antioxidant Activity of Albumin

Over the years, it has been shown that albumin has a thioesterase [92], glutathione and cysteine peroxidase [93,94] and peroxidase activity towards lipid hydroperoxides [93,94,95,96].

The authors of the work [92], by measuring the outcome of mercaptoethanol (MER), found that human blood serum contains a certain thioesterase that catalyses the hydrolysis of S-lauroylmercaptoethanol (S-LME). The authors concluded that this enzyme is serum albumin. Firstly, the unit amount of reaction product per 1 mg of crystalline HSA and per 1 mg of serum albumin was the same. Secondly, the rate of MER outcome reduced by about 50 percent with various anionic or non-ionic lauryl derivatives and urea; moreover, the product outcome terminated when HSA was inactivated with various detergents or high temperature. The rate of MER outcome significantly reduced after about 8 moles of mercaptoethanol released per mole of HSA. The authors concluded that there is an irreversible acylation of albumin amino acids. Moreover, according to the data obtained, lysines, but not tyrosines, are the most likely amino acid residues responsible for the thioesterase activity of HSA towards S-LME, since during the reaction of albumin with S-LME, no decrease in absorption at 278 nm typical for acylation of tyrosine residues was observed.

According to the Korean researchers Cha and Kim [93], a 65 kDa protein isolated from human blood plasma and identified by the *N*-terminal amino acid sequence as serum albumin was able to accelerate H_2_O_2_ reduction by GSH. The authors did not report on the kinetic characteristics of HSA for peroxide and GSH, but they noted that the rate of glutathione-dependent reduction in H_2_O_2_ in the presence of HSA in the reaction mixture was a function of the albumin concentration and had a saturation behavior. The results obtained suggested the presence of glutathione peroxidase (GSH: H_2_O_2_-oxidoreductase) activity in HSA, but the authors, unfortunately, did not report on the stoichiometry of the reaction. If the molar ratio between GSH and H_2_O_2_ in their interaction catalysed by HSA would be confirmed as 2:1, then this would allow us to more confidently say that albumin is functionally capable of being a GSH:H_2_O_2_ oxidoreductase. Later, the same group of researhers demonstrated that activation of thiol-dependent antioxidant activity of HSA at alkaline pH was due to the conformational change favorable for the functional cysteine(s)-mediated catalysis [95]. Later, it was shown that palmitoyl-CoA induced the conformational changes of HSA and thus provided thioredoxin-linked lipid hydroperoxide peroxidase activity of the protein [96].

In 1999 R. Hurst and co-authors [94] found that HSA is effective in catalysis of the reduction in 1-palmitoyl-2-(13-hydroperoxy-cis-9,trans-11-octadecadienoyl)-L-3-phosphatidylcholine to the corresponding hydroxy derivative when using thiols such as cysteine, glutathione, cysteinylglycine and homocysteine as oxidisable substrates (listed in decreasing order of their effectiveness in albumin-catalysed hydroperoxide reduction). HSA reduced phospholipid hydroperoxide in the absence of a thiol reducing agent, but at a lower rate than with any of them. The authors evaluated the stoichiometry of the reduction in the phospholipid hydroperoxide to the corresponding hydroxy derivative in the presence of albumin and cysteine. The molar ratio between the resulting 1-palmitoyl-2-(13-hydroxy-cis-9,trans-11-octadecadienoyl)-L-3-phosphatidylcholine and cystine (cysteine disulfide) was close to 1:1, which confirms the hypothesis that albumin functions as a cysteine peroxidase—i.e., catalyses the reaction according to a scheme similar to that of the glutathione peroxidase reaction: ROOH + 2Cys-SH → ROH + H_2_O + Cys-SS-Cys, where Cys-SH is cysteine, Cys-SS-Cys is cystine, ROH is a hydroxy derivative and ROOH is hydroperoxide. The kinetic characteristics towards cysteine were calculated with a fixed concentration of phospholipid hydroperoxide and vice versa, towards phospholipid hydroperoxide with a fixed concentration of cysteine [94]. The obtained values of the apparent K_m_ and V_max_ for cysteine were 600 ± 80 μM and 0.21 ± 0.02 nmol/(min × mg protein), respectively (M ± SD). The same parameters for phospholipid hydroperoxide are 9.23 ± 0.95 μM and 0.11 ± <0.01 nmol/(min × mg protein). Treatment of albumin with dithiothreitol (DTT) decreases both apparent K_m_ and increases both apparent V_max_, while modification with *n*-ethylmaleimide leads to a decrease in both K_m_ and V_max_. In general, this means that the presence of free SH-groups in the albumin molecule enhances its catalytic properties. The authors confirm the same conclusion using captopril, which increases the cysteine peroxidase activity of albumin, while the relation between activity and the concentration of captopril has a saturation behavior [94]. The results using captopril indicate the participation of Cys34 in catalysis, but, apparently, the release of additional thiol groups in the albumin molecule during DTT treatment provides greater catalytic efficiency of albumin. Surely, the cysteine peroxidase activity of albumin in relation to phospholipid hydroperoxide is low (and its glutathione, cysteinylglycine and homocysteine peroxidase activities towards the same reducible substrate are, apparently, even lower), but, as the authors fairly note, the low activity should be compensated by its high concentration in plasma [94]. Furthermore, cysteine is a major low-molecular-weight thiol in blood plasma, the physiological concentration of which is 9–12 μM [97]. The total concentration of phosphatidylcholine hydroperoxides in plasma is 20–430 nM [94]. It is probable that albumin makes a certain contribution to the catalysis of thiol-dependent reduction in phospholipid hydroperoxides in blood plasma together with other peroxidases. In any case, the presence of cysteine peroxidase (cysteine: phospholipid-hydroperoxide-oxidoreductase) activity in human serum albumin can be confidently stated. In contrast to the intracellular analogue, the monomeric Se-containing protein phospholipid hydroperoxide glutathione peroxidase (also named glutathione peroxidase-4; abbreviations—PHGPx, GPx4; EC 1.11.1.12), the role of which in the protection of cells, including nervous, from the damaging effect of lipid hydroperoxides can hardly be overestimated [98,99,100], as well as in contrast to the extracellular tetrameric glutathione peroxidase-3 (GPx3; EC 1.11.1.9), the decrease in the activity of which is consistently correlated with the development of oncological diseases [101,102], monomeric (but multi-domain) serum albumin does not contain selenium.

Paraoxonase activity of albumin is described in detail in our previous research: albumin is able to operate as a paraoxonase though does not depend on Ca^2+^ ions [72].

### 3.5. Indirect Mechanisms of Albumin Participation in the Antioxidant Defence of the Body

Roche et al. [51] discuss the ability of albumin to bind polyunsaturated fatty acids (PUFAs) and bilirubin, and thus indirectly further enhance the antioxidant defence of the body. It is known that albumin-bound bilirubin can inhibit lipid peroxidation. Bilirubin binds at Site III of albumin [103] (Figure 6). As for PUFAs, according to the authors, it is possible that in combination with albumin, they are protected from peroxidation. The amino acids Arg117, Lys351 and Lys475 are responsible for the interaction of the protein with PUFA molecules (Figure 6).

## 4. Interplay of Binding, Enzymatic and Antioxidant Properties of Albumin

As mentioned above, the structure of albumin is rather labile and tends towards allosteric modulation: binding of a ligand in one site can affect the efficiency of binding in another. Thus, the conformational changes occur in the albumin molecule after the binding of a number of endogenous compounds, such as bilirubin [104], urea [105], estradiol [106] and glucose [107]. Exogenous compounds might also have an allosteric effect. For example, the binding of lorazepam in Sudlow site II changes the binding efficiency of warfarin in Sudlow site I [108], the binding of tenoxicam in Sudlow site I enhances the binding of diazepam in Sudlow site II and vice versa [109]. These features suggest that a targeted modulation of albumin with the help of the molecules regulating its structural and functional properties can influence the process of the protein interaction with ROS and RNS. 

On the other hand, oxidative stress accompanies many diseases, the level of oxidised albumin increases, which in turn can affect the kinetics of pharmacological and toxic compounds. Therefore, it is essential to study the interaction of various activities of albumin and answer the following questions. Does oxidation of the thiol group of Cys34 (and other amino acids) affect the binding and catalytic properties of albumin towards its ligands? Does this effect depend on the oxidative agents and on the structure of the ligand? Do endogenous and exogenous compounds affect the availability and reactivity of the thiol group of Cys34 and, as a consequence, the antioxidant properties of albumin?

### 4.1. Effect of Cys34 Oxidation on the Functional Properties of Albumin

We now review the effect of Cys34 oxidation on albumin binding and pseudo(esterase) activity. Our own computational experiments, performed as a part of investigation of the interaction of albumin with OPs, were devoted to the study of the influence of the redox status of HSA on their interaction with paraoxon [73]. We tested three models of the oxidation state of albumin: Cys34 is reduced (Cys34-SH), Cys34 is oxidised to sulfenic acid (Cys34-SOH) and Cys34 is oxidised to sulfinic acid (Cys34-S(O)O^−^). According to the data obtained, the redox status of Cys34 had no significant impact on the possibility of esterase reactions at Sudlow site I. The affinity of HSA Sudlow site I does not depend on the redox status of the cysteine neither. However, the modification of the cysteine changed the conformation of Sudlow site I of HSA and the position of paraoxon molecule within the site. Oxidation of albumin practically did not affect either the conformation of the Sudlow site II of HSA, or the position of the ligand in this site or the affinity of the site for paraoxon.

Similar results were obtained in biochemical in vitro experiments with HSA. Bertucci et al. [110] showed that HSA Cys34 oxidation with ethacrynic acid did not affect the affinity of neither Sudlow site I towards phenylbutazone nor Sudlow site II towards diazepam, but improved the binding efficiency of bilirubin in the third major albumin binding site (Site III).

In the research of Anraku et al. [111], human albumin was oxidised in vitro by three different methods: by a metal-catalysed oxidation system (MCO), chloramine-T and hydrogen peroxide. It turned out that oxidation (by any means) had practically no effect on the binding of warfarin in Sudlow site I. Oxidation with hydrogen peroxide did not affect the binding of ketoprofen in Sudlow site II, but oxidation with MCO and chloramine-T reduced the affinity of Sudlow site II for ketoprofen. The different effect of different oxidants can be explained by the fact that MCO and chloramine-T can oxidise not only Cys34 but also the side chains of lysines and arginines, including Arg410 and Arg485 [111,112], localised in Sudlow site II.

The results of in vivo experiments contradict the data obtained in vitro and in silico. In healthy people, about 70% of albumin remains in a reduced form, but the level of oxidised albumin can increase in some pathological processes and during the aging process [113,114]. The research of [115] showed that albumin in patients with liver cirrhosis (a disease in which the content of oxidised albumin is increased) binds ligands of Sudlow site II more weakly than in healthy subjects. Nagumo et al. [116] revealed that the content of cysteinylated albumin (HSA-Cys34-S-S-Cys) increased in patients with chronic kidney and liver disease. The binding activity of albumin towards warfarin (a ligand of Sudlow site I) and diazepam (a ligand of Sudlow site II) in these patients was significantly lower than in healthy people. Thus, the modification of Cys34 impaired the affinity of Sudlow sites I and II for both warfarin and diazepam, which contradicts in vitro data [110,111]. 

There are several possible explanations for the conflict between in vitro and in vivo data. One of them is that liver and kidney disease can lead to increased levels of certain molecules in blood plasma, which in turn can inhibit (competitively or non-competitively) the binding of ligands in Sudlow sites. For example, it is known that the level of glucose in blood can be increased in liver cirrhosis [117]. On the other hand, the authors of [118] showed that oxidation of albumin SH-groups with potassium permanganate led to an increase in the number of the sites on the albumin surface available for glucose binding. One more explanation is that serum albumin is loaded with fatty acids (FAs) in blood [119], which can affect the binding characteristics of the protein in both reduced and oxidised form. 

### 4.2. Effect of Cys34 Oxidation on the Structural Properties of Albumin

Changes in the functional characteristics of albumin caused by a change in its redox status are primarily the result of the structural rearrangements in the protein molecule. A number of spectroscopic studies have been carried out so far to study the structural characteristics of reduced and oxidised albumin.

For example, Maciążek-Jurczyk and Sułkowska [120] studied how the oxidation of HSA with chloramine-T affects the spectral characteristics of the protein. The method of synchronous fluorescence spectroscopy revealed changes in the position of the fluorescent band of the tyrosines and of tryptophan Trp214 (the single tryptophan in the HSA molecule) in oxidised albumin compared to the native protein. The red-edge shift spectroscopy technique demonstrated chloramine-T-induced structural changes in the environment of Trp214 located in the immediate vicinity of Sudlow site I. It was confirmed by proton nuclear magnetic resonance (^1^H NMR) that the oxidation of HSA led to structural changes in the protein molecule, mainly in the surrounding of Cys34 and Trp214. Moreover, the data obtained indicated structural changes in the conformation of the peptide backbone.

It is interesting to compare the study of Maciążek-Jurczyk and Sułkowska with the paper of Sakurama et al. [112], who also oxidised HSA with chloramine-T and studied the conformational changes in the protein molecule by the circular dichroism method. This research did not reveal significant structural change in oxidised HSA. In both studies, chloramine-T and HSA were mixed in similar proportions at the same temperature. The possible explanations are that different albumin samples and different ways to interrupt the oxidation reaction were used in these experiments.

Despite some disagreements regarding the conformational rearrangements of the albumin polypeptide chain after oxidative modification, oxidation of albumin definitely leads to the conformational changes of amino acids in the microenvironment of the modification sites.

In the research of Pieniazek et al. [121], albumin was isolated from the plasma of healthy volunteers and patients with chronic kidney disease (CKD) on hemodialysis. It was demonstrated by the method of electron paramagnetic resonance (EPR) that oxidation of albumin of the healthy subjects with hydrogen peroxide and tert-butyl hydroperoxide led to conformational changes in the microenvironment of the binding sites of maleimide and iodoacetamide spin labels, which interact predominantly with the thiol group of Cys34. The oxidants practically did not affect the structural characteristics of albumin from plasma of the subjects with CKD, since albumin of these patients had been already significantly oxidised. 

Christodoulou et al. [122] reduced fatty acids free BSA with DTT and oxidised with auranofin, and then studied the structural features of the samples by ^1^H NMR. Comparing the spectra, the authors suggested that the oxidation of Cys34 led to a change in the conformation of His3 at the *N*-terminal site of the protein.

**Figure 7:**
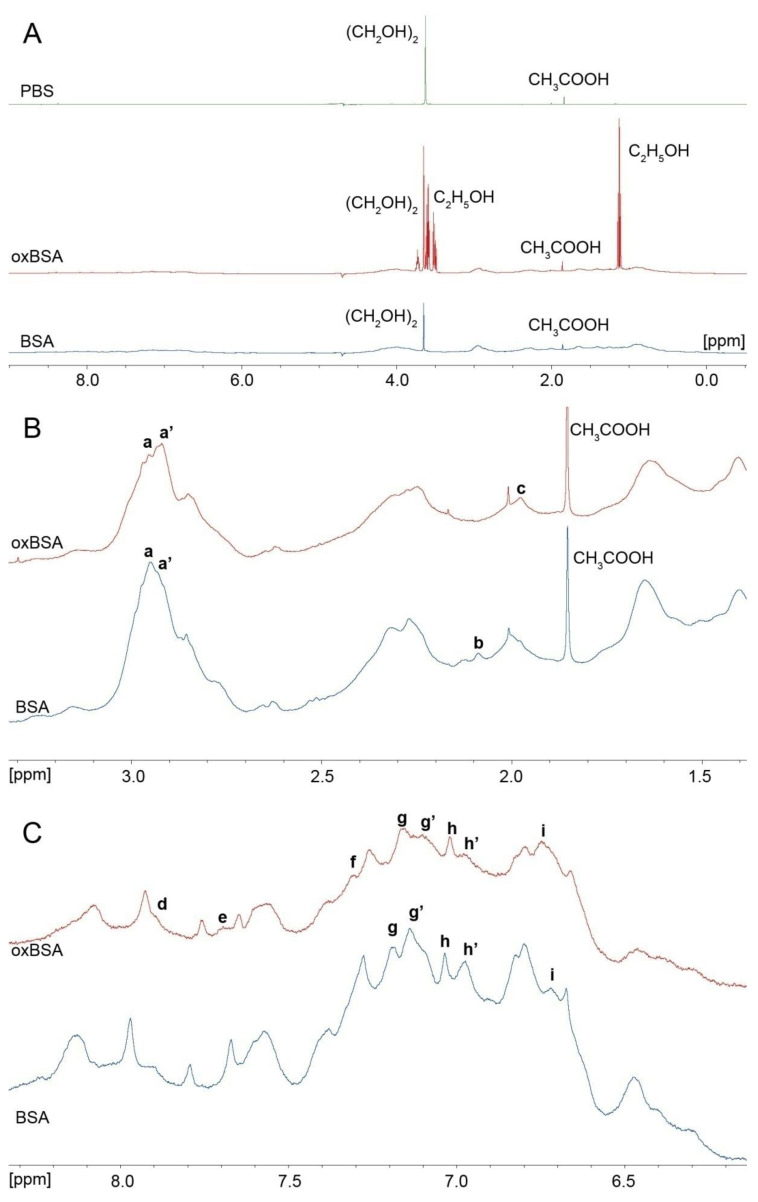
^1^H NMR spectrum of phosphate buffered saline (PBS, green), commercial bovine serum albumin (BSA, blue) and BSA incubated with ethacrynic acid in a molar ratio of 1:5 (oxBSA, red). (**A**) Full spectrum. (**B**) Aliphatic region. (**C**) Aromatic region.

In our own studies, we have applied the ^1^H NMR method to study how the oxidation of BSA with ethacrynic acid (EtAc) affects the conformational characteristics of the protein. Figure 7 shows the spectra of three samples: phosphate buffered saline (PBS) used to prepare BSA solution; commercial BSA of concentration 360 μM; commercial BSA of concentration 360 μM after incubation with EtAc in a molar ratio of 1:5 (oxBSA). Commercial BSA was prepared using the same procedure as in [123]. Oxidised BSA was prepared as described in [110] with minor modifications. The prepared samples of commercial and oxidised BSA were supplemented with deuterium oxide and scanned at room temperature by the one-dimensional ^1^H-NMR water suppression method using excitation sculpting with gradients on a Bruker Avance III 500 NMR spectrometer. Chemical shifts δ were calibrated to tetramethylsilane; the spectra were accumulated for 128 scans using a 4.3 s delay between the first radiofrequency pulses.

Figure 7A shows the full spectrum. Both samples of BSA contain the impurities associated with the imperfect purity of the supplied PBS tablets (green spectrum). Based on the literature, it is highly likely that the peak with a chemical shift of 1.85 ppm corresponds to acetic acid (CH_3_COOH) [124,125] and a singlet with a chemical shift of 3.6 ppm most likely corresponds to ethylene glycol (CH_2_OH)_2_ [126,127,128]. Additionally, the sample of oxidised BSA contains ethanol (C_2_H_5_OH), which was used to dissolve EtAc.

Figure 7B shows the aliphatic region of the spectrum. The change in the shape of the spectrum in the region 3.1–2.8 ppm (peaks **a** and **a’**) can probably be associated with a change in conformation of the microenvironment of Cys34 (C_β_H_2_ groups [129]) after its oxidation with EtAc. Differences between the two samples can also be observed in the region 2.08–1.98 ppm (peaks **b** and **c**). We suppose that this might be due to a change in the conformation of glutamine Gln33 (signal of the C_γ_H_2_ group [130,131]) and/or proline Pro35 (signal of the C_γ_H_2_ group [130,131]) located in the microenvironment of Cys34 (Figure 5B). According to the literature, Cys34 oxidised to sulfenic or sulfinic acids can form an intramolecular bond with Gln33, while these amino acids do not interact in reduced albumin [87].

Figure 7C shows the aromatic region of the spectrum. The change in the shape of the spectrum in the regions 8.2–7.5 ppm and 7.1–6.9 ppm reflects the change in signals from C_ε_H and C_γ_H_2_ groups of histidines, respectively [128,129,132]. The appearance of a weak signal **d** and a decrease in the intensity of **h’** peak in oxBSA probably indicates a change in the conformation of His39, which interacts with the SH-group of Cys34 in reduced but not in oxidised albumin [73,74]. Stewart et al. also noted the importance of His39 in the reactivity of the thiol group of Cys34 [133]. 

As we have mentioned above, in [122], a change in the signal in this region after BSA oxidation was proposed to be due to a change in the conformation of His3 in the NTS of albumin. So, we think that peak **e** in oxBSA can correspond to a change in His3 conformation. 

The region at 7.3–6.6 ppm corresponds to the signals of the aromatic rings of tyrosine residues [128,129,132]. The change in the shape of the spectrum in this region (peaks **g**, **g’** and **i**) is highly likely associated with a change in the conformation of Tyr84 and its microenvironment. According to abundant evidence, Tyr84 plays a key role in the reactivity of Cys34 [133,134]. Additionally, we suppose that peak **f** in oxidised BSA could be a signal of the benzene ring of EtAc covalently bound to the SH-group of Cys34 [135]. The signal of the second aromatic hydrogen of EtAc in oxBSA might contribute to the intensity of peak **g** too.

Thus, according to the data obtained, BSA oxidation leads to a change in the conformation of the microenvironment of Cys34: Gln33, Pro35, His39 and Tyr84. It should be mentioned that it is undoubtedly difficult to unambiguously interpret the one-dimensional NMR spectrum of such a composite protein as albumin. Our conclusions are rather in the nature of an assumption, but nevertheless the result is in fairly good agreement with the literature data. 

The tools for studying the structural characteristics of macromolecules are constantly evolving. Thus, the solution structure (which is more natural than the crystal one) of some proteins with a molecular weight over 30 kDa have been obtained by NMR technique to date [136]. In future, it probably would be possible to obtain the solution structure of albumin, the molecular weight of which is 66 kDa, and to trace how the structure of the protein changes when interacting with various ligands. Molecular modeling methods are being developed, too: the mathematical apparatus describing the interaction of atoms is being improved; computer power is growing. Currently, the classical molecular dynamics is the main computer method for studying the conformational changes of macromolecules; however, it cannot simulate the changes in the structure of a protein at atomic level (for example, the transfer of a proton from one amino acid to another or the formation of covalent bonds between ligands and proteins). With the development of computing power, it became possible to apply the method of quantum molecular dynamics, which is able to simulate these processes [137]. Additional spectroscopic and computational experiments will help amplify the obtained information about structural rearrangements in the albumin molecule after oxidation or reduction in Cys34 in future. 

### 4.3. Effect of Endogenous and Exogenous Compounds on the Reactivity of the Thiol Group of Cys34

Now, we consider the possibility of modulating the antioxidant properties of albumin. First of all, it is obvious that the oxidation of the thiol group of Cys34 or its nitrosylation (Cys34-S-N=O) reduces the ability of albumin to neutralise ROS and RNS [85]. 

However, in addition to the direct oxidation of Cys34, albumin can undergo other chemical modifications that affect its structure and conformation, which in turn can lead to modulation of its antioxidant properties. Glycation is one of these modifications, which is the covalent binding of glucose or another monosaccharide to the side chains of lysines and arginines [138]. To date, more than 60 albumin glycation sites have been described, but many researchers agree that Lys525 is the most reactive of them [139,140,141]. Modifications caused by glycation have an important effect on the functional properties of albumin, mainly associated with the changes in its conformation [142,143,144,145].

However, FAs appear to play the main role in the regulation of the antioxidant properties of albumin. For the first time, this conclusion was made by Gryzunov and co-authors [80,146]. According to the data obtained, blocking of Cys34 by *n*-ethylmaleimide did not affect the fluorescence intensity of probe K-35 (binding in the Sudlow site I) in HSA free of FAs [80]. However, adding FAs (oleic and linoleic), firstly, changed the conformations of Sudlow sites I and II, and, secondly, strengthened the reactivity of Cys34 thiol group towards 5,5′-dithiobis-2-nitrobenzoic acid (DTNB) having increased its steric availability. The authors hypothesised that FAs, when bound to albumin, simultaneously regulated both its transport and antioxidant functions, serving as a necessary intermediary between these activities [146].

A similar result was obtained in the research of Torres et al. [147]. The authors showed that in the presence of FAs (palmitic, myristic, lauric, stearic, oleic), the reactivity of HSA towards DTNB increased by about 6 times (with a minor scatter depending on FA structure) compared to FAs free albumin. Stearic acid doubled the rate of the reaction of Cys34 with hydrogen peroxide and peroxynitrite and strengthened the reactivity of sulfenic acid of HSA towards low-molecular-weight thiols. Oxidation of Cys34 thiol group did not change the efficiency of the interaction of HSA with FAs.

Pavićević et al. [148] showed that the binding of FAs (palmitic, docosahexaenoic, stearic, oleic, myristic, eicosapentaenoic and fish oil) with HSA increased the reactivity of Cys34 towards methylglyoxal. Subsequent experiments demonstrated that the reaction of DTNB with Cys34 (both reduced and modified with methylglyoxal) was accelerated in the presence of fatty acids too. The same research group demonstrated later that the binding of polyphenols enterolactone and enterodiol with HSA increased the reactivity of the Cys34 SH-group towards DTNB [149]. It was shown that fatty acids were able to modulate this effect. Finally, one of the latest investigations of this group revealed that the binding of copper cations Cu(II) with defatted HSA practically did not affect the reactivity of Cys34, while the addition of copper to the complex of HSA with FAs (oleic, myristic, or fish oil) increased the reactivity of the cysteine cumulatively [150].

**Figure 8:**
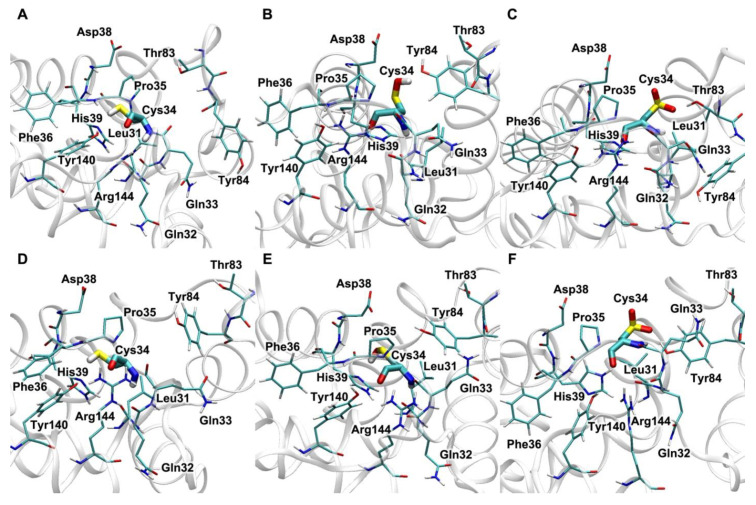
Effect of paraoxon on the conformation of human serum albumin redox site with different oxidation states of Cys34 according to molecular modeling data. (**A**–**C**)—paraoxon is bound in Sudlow site I; (**D**–**F**)—is bound in Sudlow site II; (**A**,**D**)—Cys34 is reduced (Cys34-SH); (**B**,**E**)—Cys34 is oxidised to sulfenic acid (Cys34-SOH); (**C**,**F**)—Cys34 is oxidised to sulfinic acid (Cys34-S(O)O^−^).

In our recent computational experiments [73], we have analysed how the redox status of HSA affects the binding of paraoxon in Sudlow sites I and II. However, an analysis of the effect of paraoxon binding on the conformation of Cys34 and its microenvironment has not been performed. Here we fill this gap. Figure 8 shows how the conformation of Cys34 with different oxidation level of the thiol group depends on the occupancy of Sudlow sites. In the upper row (Figure 8A–C), paraoxon is bound in Sudlow site I; in the lower one (Figure 8D–F), it is bound in Sudlow site II. 

It could be noticed that the occupancy of Sudlow sites has the greatest effect on the conformation of Cys34 oxidised to sulfenic acid (Cys34-SOH) (Figure 8B,E). When paraoxon is bound in Sudlow site I (Figure 8B), the availability of the -SOH group is greater than in the case of paraoxon bound in Sudlow site II. It has been mentioned in Section 3.2 that HSA-Cys34-SOH is a central intermediate in the processes of redox modulation of blood plasma and intercellular fluid, and the final result of the oxidative process depends on what happens with this sulfenic acid. However, our observation has more theoretical than practical significance, since the concentration of the lethal dose of paraoxon in blood hardly exceeds 15 μM, which can in no way affect the total albumin pool.

Recently, Litus et al. performed multifactorial computational disorder analysis of HSA and BSA [151]. For all the residues of HSA and BSA, the authors calculated the values of mean predicted disorder scores (MPDSs) characterising the flexibility of the amino acids, and then they analysed the MPDS values of the phosphorylation, acetylation, ubiquitination, methylation and glycosylation sites. According to the data obtained, serum albumins in their function (including the antioxidant properties) often rely on disordered or flexible residues characterised by MPDS ≥ 0.5 and 0.2 ≤ MPDS < 0.5, respectively. For example, the arginines and lysines participating in albumin glycation are characterised by rather high disorder scores ranging from 0.19 to 0.53. The amino acids of NTS Asp–Ala–His–Lys (binding site for Cu(II)) have MPDS values of 0.31, 0.31, 0.32 and 0.36, respectively. The researchers concluded that intrinsic disorder and high structural flexibility are important for the functionality of serum albumin.

In conclusion of this section, it can be noted that all the papers mentioned above indicate the fundamental possibility of modulating the antioxidant properties of albumin with endogenous and exogenous ligands. Another important aspect worth paying attention to is that FAs are the key transmitters of information between the sites of binding and antioxidant activity of albumin. This fact must be taken into account in the biochemical studies of the drugs interacting with albumin.

## 5. Antioxidant Properties of Albumin: Practical Application

Albumin is usually one of the first proteins to be influenced oxidative stress; therefore, its redox status is widely used as a biomarker of various pathological conditions. It is known that in chronic liver and kidney diseases, as well as in diabetes mellitus, the percentage of cysteinylated albumin (Cys34-S-S-Cys) is markedly increased [116]. In recent years, it has been shown that oxidised albumin can be a biomarker of the severity of such diseases as hyperparathyroidism [152], acute ischemic stroke [153], Parkinson’s disease [154], Alzheimer’s disease [155], Duchenne muscular dystrophy [156], etc.

The possibility of using the covalent binding of the products of OPs hydrolysis with Cys34 for developing the biomarkers of intoxication is of particular interest. OPs adducts with Tyr411 are widely studied and described in the literature [157,158,159]. However, for thioether OPs (such as VX), another class of adducts can be identified. Kranawetvogl et al. [160] showed that the thiol formed after hydrolysis of this class of OPs can interact with the thiol group of Cys34, and the resulting adduct can be detected by mass spectroscopic methods. The same group of researchers, in a recent work [161], studied a real case of poisoning with demeton-S-methyl (*O*,*O*-Dimethyl *S*-2-(ethylsulfanyl)ethyl phosphorothioate, ODM). ODM belongs to the class of dimethylphosphoryl (DMP) pesticides. 2-(ethylsulfinyl) ethanethiol (ESOET) is a product of ODM hydrolysis by blood esterases. The patient’s blood plasma was treated with pronase (a mixture of proteinases), and then the obtained samples were examined by mass spectroscopy. Among the identified adducts of albumin with ODM, the adduct DMP-Tyr (Tyr411) had the weakest peak, and could only be detected within two hours after poisoning. The peak intensity of the ESOET-CysPro adduct (Cys34 and Pro35 of albumin) was 200 times higher, and its lifetime was 73 h.

Fujii et al. [162] performed a comprehensive study of 281 Japanese residents: the ratio of oxidised/reduced albumin, the thickness of the intima-media complex of the carotid arteries and the number of plaques in the carotid arteries (the latter two indicators characterise the risk of atherosclerosis) were measured. An inverse relationship was found between the level of oxidised albumin and the risk of atherosclerosis. Violi et al. recently showed that HSA level is independently associated with mortality in COVID-19 [163]. The researchers suggested that it might be connected with the antioxidant and anticoagulant properties of albumin.

Attempts are being made to use albumin not only as an informant about the condition of patients but also as a therapeutic agent. An interesting application of the redox properties of albumin was proposed by Japanese scientists [164]. It is known that reactive sulfur species (RSS) are able to neutralise ultraviolet radiation products (for example, ROS and NO) that promote melanin synthesis. However, the instability of RSS limits their use as inhibitors of melanin synthesis. The authors proposed a method for using albumin as the RSS delivery system. It was shown that thiolated albumin (obtained by the incubation of albumin and sodium polysulfide) significantly inhibited melanin synthesis in B16 melanoma cells. The researchers also suggested that albumin modified in such a way could be used in cosmetology to whiten the skin.

In the research of Schneider et al. [165], the possibility of using human albumin solution to protect patients of an intensive care unit (ICU) from bacterial infections was studied. The polypeptide vasostatin-1 is known to have antimicrobial properties and play a key role in protecting the body from gram-positive bacteria. However, the oxidised form of vasostatin loses its antibacterial properties. Oxidative processes are often developed in ICU patients, which means that they are more at risk of infection. The study showed that continuous infusion of 4% albumin reduced the risk of nosocomial infections. By mixing albumin with oxidised vasostatin-1 and using a high-performance liquid chromatography (HPLC) method, the authors demonstrated that albumin reduced the oxidised form of vasostatin, thereby increasing its antibacterial properties.

Analysis of the literature data allows us to take a fresh look at the results of our research aimed at the development of adjuvant therapy for OP poisoning. In Section 4.3, we have reviewed studies demonstrating that many FAs and some polyphenols affect the reactivity of HSA Cys34 thiol group. Earlier in our experiments, we tested polyphenols of green tea extract (GTE) as a component of functional nutrition before and after acute poisoning with paraoxon and demonstrated the weakening effect of GTE on the development of delayed symptoms of poisoning [166]. In biochemical in vitro experiments, we have shown that GTE polyphenols have an activating effect on the true esterase activity of the protein in Sudlow site I towards paraoxon [167] and have suggested that GTE promotes not only the transport but also the utilisation of OPs by albumin in the bloodstream. However, in the light of new data, it is possible that the major polyphenol of GTE epigallocatechin gallate (EGCG) has an additional effect: by binding to albumin, it affects the reactivity of the Cys34, enhances its antioxidant properties, weakens the strength of oxidative stress and thereby reduces the intensity of delayed effects of poisoning. This hypothesis requires additional testing.

Despite some progress in studying the possibility of enhancing the antioxidant properties of albumin, the development of the methods for correcting oxidative stress taking into account this ability of the protein is still in its infancy. Recently, such classes of compounds as thiol antioxidants (*n*-acetylcysteine, carbocysteine and erdosteine), superoxide dismutase mimetics (magnesium-containing porphyrins), NADPH oxidase inhibitors (apocinin, diphenyliod), setanaxib traps (disulfenton sodium), activators of the transcription factor Nrf2 (Sulforaphane, Bardoxelone methyl, Dimethylfumarate) have been actively tested or are already being used to reduce oxidative stress [168,169,170,171]. It might be that in the future it will be possible to create the complex therapy for oxidative stress management taking into account the functional properties of albumin.

## 6. Concluding Remarks

The literature data analysed and the results of our own research allow us to formulate some concluding remarks. Firstly, albumin is a universal molecule in a certain sense, which can bind almost all known endogenous compounds, metal ions and xenobiotics and possesses a number of enzymatic activities: (pseudo)esterase, paraoxonase, phosphotriesterase, thioesterase, glutathione peroxidase, cysteine peroxidase and some others. Due to this versatility, albumin is a participant of many biochemical processes in the human organism, including participation in antioxidant defence. Of course, albumin takes part in the redox reactions non-specifically due to the fact that its concentration in the extracellular compartment is very high and renewal occurs relatively quickly (about 20 days). At the same time, it is a sacrificial antioxidant, which takes the brunt of the extracellular component of oxidative stress. The second important point to note is that albumin is easily modulated due to its flexible structure. The interaction of albumin with active species and oxidation of Cys34 can lead to an alteration of the protein binding properties towards the ligands, in particular pharmaceuticals and toxic substances. Additionally, the binding of some compounds affects the reactivity of the thiol group of Cys34 and modulates the antioxidant properties of the protein in the direction of strengthening or weakening. Undoubtedly, these properties of albumin should be taken into account in the development of therapy for pathologies associated with oxidative stress.

## Abbreviations

^1^H NMR	Proton nuclear magnetic resonance
ACh	Acetylcholine
AChE	Acetylcholinesterase
BH4	Tetrahydrobiopterin
BSA	Bovine serum albumin
CaM	Calmodulin
CAT	Catalase
CKD	Chronic kidney disease
COVID-19	Coronavirus Disease 2019
CYP450	Cytochrome-dependent oxygenases
DHPR	Dihydropyridine receptors
DMP	Dimethylphosphoryl
DTNB	5,5′-dithiobis-2-nitrobenzoic acid
DTT	Dithiothreitol
EGCG	Epigallocatechin gallate
eNOS	Endothelial nitric oxide synthase
EPR	Electron paramagnetic resonance
ESOET	2-(ethylsulfinyl) ethanethiol
EtAc	Ethacrynic acid
ETC	Electron transport chain
EtOH	Ethanol
FAs	Fatty Acids
GPx	Glutathione peroxidase
GPx3	Tetrameric glutathione peroxidase-3
GR	Glutathione reductase
Grx	Glutaredoxins
GSH	Reduced glutathione
GSSG	Oxidised glutathione
GST	Glutathione-S-transferase
GTE	Green tea extract
HPLC	High-performance liquid chromatography
HSA	Human serum albumin
ICU	Intensive care unit
iNOS	Inducible nitric oxide synthase
LCAD	Long-chain fatty acid dehydrogenase
MBS	Metal-binding site
MCO	Metal–catalysed oxidation system
MPDS	Mean predicted disorder score
MER	Mercaptoethanol
MM-PBSA	Molecular Mechanics/Poisson-Boltzmann Surface Area
nNOS	Neuronal nitric oxide synthase
NOS	Nitric oxide synthase
NOX	NAD(P)H-oxidase system
NPA	p-nitrophenyl acetate
NTS	*N*-terminal site
ODM	O,O-Dimethyl S-2-(ethylsulfanyl)ethyl phosphorothioate
OPs	Organophosphates
oxBSA	Oxidised bovine serum albumin
PBS	Phosphate buffered saline
PHGPx	Phospholipid hydroperoxide glutathione peroxidase
PON	Paraoxonase
Prxs	Peroxiredoxin
PUFAs	Polyunsaturated fatty acids
RNS	Reactive nitrogen species
ROS	Reactive oxygen species
RSA	Rat albumin
RSS	Reactive sulfur species
RyR	Ryanodine receptors
S-LME	*S*-lauroylmercaptoethanol
SOD	Superoxide dismutase
SR	Sarcoplasmic reticulum
TR	Thioredoxin reductase
Trx	Thioredoxin
VLCAD	Very long-chain fatty acid dehydrogenase
XO	Xanthine oxidase
